# The association of polymorphisms of *TLR4* and *CD14* genes with susceptibility to sepsis in a Chinese population

**DOI:** 10.1186/s12881-014-0123-4

**Published:** 2014-11-14

**Authors:** Haiyan Wang, Yesheng Wei, Yi Zeng, Yueqiu Qin, Bin Xiong, Gang Qin, Jun Li, Donghai Hu, Xiaowen Qiu, Suren R Sooranna, Liao Pinhu

**Affiliations:** Intensive Care Medicine, Affiliated Hospital of Youjiang Medical University for Nationalities, No.18 Zhongshan Road II, Baise, 533099 Guangxi PR China; Intensive Care Unit, the People’s Hospital of Guangxi Zhuang Autonomous Region, 6 Taoyuan Road, Nanning, 530021 Guangxi PR China; Intensive Care Unit, Minzu Hospital of Guangxi Zhuang Autonomous Region, 323 Mingxiu East Road, Nanning, 530021 Guangxi PR China; Department of Surgery and Cancer, Imperial College London, Chelsea and Westminster Hospital, London, SW10 9NH UK

**Keywords:** Sepsis, Single nucleotide polymorphism, *TLR4*, *CD14*

## Abstract

**Background:**

Sepsis is now the leading cause of death in the non-cardiovascular intensive care unit (ICU). Recent research suggests that sepsis is likely to be due to an interaction between genetic and environmental factors. Genetic mutations of toll-like receptor 4 (*TLR4*) and cluster of differentiation 14 (*CD14*) genes are involved in the immune and (or) inflammatory response. These may contribute to the susceptibility to sepsis in patients. This study was designed to evaluate whether the *TLR4* and cluster *CD14* gene polymorphisms are associated with susceptibility to sepsis.

**Methods:**

The single nucleotide polymorphisms (SNPs) of *TLR4* (rs10759932, rs11536889, rs7873784, rs12377632, rs1927907, rs1153879) and *CD14* (rs2569190 and rs2563298) in patients with sepsis and control subjects in the Guangxi Province were analyzed by using the polymerase chain reaction-single base extension (PCR-SBE) and DNA sequencing methods.

**Results:**

The rs11536889 polymorphism in *TLR4* and rs2563298 polymorphism in *CD14* were significantly associated with the risk of sepsis when compared to the control group. The frequencies of rs11536889 and rs2563298 polymorphisms in the group with sepsis were higher than that in the control group (*OR* = 1.430, 9*5% CI*, 1.032-1.981, *P<*0.05; *O*R = 2.454, *95% CI*, 1.458-4.130, *P*<0.05, respectively). Followed up haplotype analysis suggested that there were two haplotypes in which increased risk factors for sepsis were indicated.

**Conclusions:**

The rs11536889 polymorphism in *TLR4* and rs2563298 polymorphism in *CD14*, and two haplotypes were associated with increased susceptibility to sepsis.

## Background

Sepsis is a clinical, catastrophic condition whereby pathogenic microorganisms are able to trigger in the host, the immune and coagulation systems as well as apoptosis which result in systemic inflammatory response syndrome (SIRS). In response to infection, increased amounts of proinflammatory and antiinflammatory mediators are released. The imbalance between proinflammatory and antiinflammatory effectors may lead to cell and tissue injury as well as organ function disorder, which can results in a series of serious medical complications [1].

Despite significant advances in intensive care, improved supportive measures and popularized evidence-based guidelines, the prognosis for sepsis is still not ideal, and severe sepsis and septic shock are also the leading causes of death in the non-cardiovascular intensive care unit, even in developed countries [2].

Patients with similar general clinical symptoms and infected with the same microorganisms and undergoing similar therapy could present with different clinical outcomes, which indicate that genetic factors may be involved in these processes [3,4]. Research evidence suggests that sepsis is likely to be due to an interaction of genetic and environmental factors [5-7]. Genetic factors may affect the body’s response to infection caused by microorganisms. Genetic mutation in genes involved in the immune and (or) inflammatory response may contribute to the susceptibility to sepsis [6]. This may explain the clinical variability observed during similar infections. The infection-triggered systemic inflammatory response plays a critical role in the pathogenesis of sepsis [8,9]. The immune response is the first-line response to defense microbial infections. Pattern recognition receptors (PRRs) are essential for triggering the immune response and these include the *TLR* gene family. These receptors can recognize molecules expressed on the surface of microorganisms, which are called pathogen-associated molecular patterns (PAMPs). It is postulated that SNPs in these receptors may influence their ability to recognize microorganisms [10].

*TLRs* are an evolutionarily conserved family of receptors which play fundamental roles in pathogen recognition and the innate immune and inflammatory response [11-14]. *TLRs* have three functional domains within their structures: an ectodomain, which contains multiple leucine-rich repeats (LRR) participating in the recognition and binding of pathogens, a cytoplasmic domain that also spans the membrane and a toll/interleukin-1 receptor (TIR) domain. To date thirteen members of the *TLR* family have been identified [15]. *TLR4* is one of the *TLR* family members that has been the widely most investigated. The gene encoding for human *TLR4* is mapped on chromosome *9q32-33* and includes 3 exons and 2 introns. *TLR4* can recognize a variety of pathogens, including Gram-negative and Gram-positive bacteria [16], fungi [17], viruses [18] and protozoa [19]. *CD14* is a coreceptor that contributes to *TLR*-induced cell activation. Upon binding to the microbial ligands, *CD14* becomes linked to *TLR4* and then, *TLR4* can activate downstream signaling transduction pathways, such as the nuclear factor-kappa B (NF-κB) signaling pathway. This subsequently mediates the production of cytokines, chemokines and coagulation factors which are involved in the pathological process of inflammation [12,20].

It has been reported that some polymorphisms in the *TLR4* and *CD14* genes may regulate their expression, thereby, influencing the production of *TLR4* [21] and *CD14* [22,23]. Several studies have shown that polymorphisms in *TLR4* and *CD14* genes may relate to the susceptibility with some infection induced diseases, e.g. sepsis [24-28], but this is controversial.

In this study, we investigated the relationship between sepsis and *TLR4* gene rs10759932, rs11536889, rs7873784, rs12377632, rs1927907 and rs1153879 polymorphisms as well as *CD14* gene rs2563298 and rs2569190 polymorphisms in a Chinese population. Genotyping analysis of eight SNPs in *TLR4* and *CD14* genes were performed by using Snapshot SNP genotyping assays and DNA sequencing methods to investigate whether the gene polymorphisms are associated with susceptibility to sepsis.

## Methods

### Study subjects

The clinical characteristics of the study subjects are shown in Table 1. One hundred and fifty-two patients (48 females and 104 males) from the ages of 18 to 80 years (average age-55.47 ± 16.48) with sepsis were recruited for this study between July 2011 and December 2012 in the ICU of the Affiliated Hospital of Youjiang Medical University for Nationalities, Guangxi, PR China. The inclusion criteria were according to the American College of Chest Physicians/Society of Critical Care Medicine (ACCP/SCCM) criteria for sepsis, severe sepsis, or septic shock. Exclusion criteria were: 1. patients younger than 18 or older than 80 years old; 2. cardiac arrest; 3. emergency surgery; 4. receiving an immunosuppressive therapy. In addition, patients from whom consent could not be obtained were excluded from the study.

**Table 1:** **The clinical characteristics of the study subjects**

**Parameters**	**Cases (n = 152)**	**Controls (n = 199)**
Age	55.47 ± 16.48	53.93 ± 14.45
Male	104	118
Female	48	81
Site of infection		
Lung	80	
Abdomen	51	
Blood	10	
Undefined site	11	
Co-morbidities		
Hypertension	20	
Diabetes	15	
Renal dysfunction	5	
Liver dysfunction	4	
ARDS	6	
COPD	7	
Sepsis	17	
Severe sepsis	115	
Septic shock	20	
APACHEII	20.5 ± 6.4	

The control subjects underwent a routine medical check-up in the outpatient clinic of the Department of Internal Medicine, Affiliated Hospital of Youjiang Medical University for Nationalities, Guangxi, China between July 2011 and December 2012. According to the thorough clinical and laboratory evaluation, none of them was found to have any medical condition other than infection, history of cardiac arrest or receiving an immunosuppressive therapy. One hundred and ninety-nine control subjects from similar ethnic background, age and gender (81 females and 118 males, aged between 25 and 80 years) were also studied. The protocol used for the study was approved by the Local Ethical Committee of Youjiang Medical University of Medical Sciences, and written informed consent was obtained from all participants. All participating subjects were of Guangxi origin.

### DNA extraction and PCR assay

5 mL of venous blood was collected from each patient for genetic studies. Genomic DNA was extracted from whole peripheral blood using a QIA Amp DNA Blood Mini Kit (Qiagen, Germany) according to the standard protocols. The DNA was subsequently stored at −20°C until needed. Before use, the DNA was resolved using a 1% agarose gel stained with the ethidium bromide.

The following sequences obtained from GenBank were used as reference sequences for *TLR4* (Gene ID: 7099): NG_011475.1: 4994–18310 (genomic) and *CD14* (Gene ID: 929): NG_023178.1: 5001–6974 (genomic). PCR primers were designed using Primer 3 Input (version 0.4.0; Table 2). The PCR reactions consisted of 1x HotStart Taq buffer, 3.0 mM magnesium chloride, 0.3 mM dNTP mixture, 1 U HotStart Taq polymerase, forward and reverse primer mixtures and genomic DNA. The PCR conditions were as follows: 95°C for 2 min, followed by 11 cycles of: 94°C for 20 sec, annealing temperature depending on the primer, 72°C for 90 sec, 24 cycles of: 94°C for 20 sec, 59°C for 30 sec, 72°C for 90 sec, and then 72°C for 2 min followed by 4°C until the reaction mixtures were removed from the cycler.

**Table 2:** **The primer sequences used for detecting the different TLR4 and CD14 SNPs**

**SNP ID**	**PCR primers**
**TLR4**	
rs10759932	F: 5'-TGCAAGCTTCTGCTATGATTAAAAGTGAT-3'
	R: 5'-TCATGGACACTTGCATTGTTGC-3'
	EF: 5'-TTTTTTTTTTTTTGAGTTCTCATTTTTTCACATCTTCACCAAC-3'
rs12377632	F: 5'-TCCCCAGGGTCTATTTTTGTCATC-3'
	R: 5'-GGGAAGCTGGCCTCTCTGTAAGC-3'
	EF: 5'-TTTTTTTTTTTTTTTTCAAGTACTCTATTAAGGTAGACCACCTCTCCC-3'
rs1927907	F: 5'-TCCCCAGGGTCTATTTTTGTCATC-3'
	R: 5'-GGGAAGCTGGCCTCTCTGTAAGC-3'
	EF: 5'-TTTTTTTTTTTTTTTTTTTTTGAAGATGAATTACATAAGAGACATTGTTTR-3'
rs11536879	F: 5'-CCTGTTGGGGTCAGAAGACCTG-3'
	R: 5'-TCGATTGTACCCTACACCTCAGCATTA-3'
	EF: 5'-TTTTTTTTTTTTTTTTTTTTTATAAGTTTCATCATTTCCATTGATCAGATA-3'
rs11536889	F: 5'-GCTGGGATCCCTCCCCTGTA-3'
	R: 5'-TGGGAACCTTCTTTATAAGAACCCCATTA-3'
	EF: 5'-TTTTTTTTTTTTTTTTTTTTTTTTTTTTTTTTTTGCTCCTTGACCACATTTTGGGAA-3'
rs7873784	F: 5'-GGTTCCTAGGGAAAAGGAGGAAGG-3'
	R: 5'-CATCACCTCCAAAAGCTTCCTTG-3'
	EF: 5'-TTTTTTTTTTTTTTTTTTTTTTTTTTTTTTTTTAGCTCTAAAGATCAGCTGTATAGCAGAGTTY-3'
**CD14**	
rs2569190	F: 5'-TCTTCGGCTGCCTCTGACAGTT-3'
	R: 5'-TTTTCCCACACCCACCAGAGAA-3'
	EF: 5'-TTTTTTTTTTTCCTGCAGAATCCTTCCTGTTACGG-3'
rs2563298	F: 5'-TGAATTCCCCATCCAGCACTGT-3'
	R: 5'-CTTCCTGGTCCCTGGAACTGC-3'
	EF:5'-TTTTTTTTTTTTTTTTTTTTTTTTTTTTTTTTTTTTTCCCCACCTTTATTAAAATCTTAAACAACGG-3'

F: forward, R: reverse, E: extension.

### Genotyping procedure

PCR products were sequenced using the ABI PRISM SNaPshot Multiplex Kit according to the protocols. Detection and sequencing were carried out with a 3730XL ABI Genetic Analyzer. Results were analyzed using GeneMapper 4.1 (Applied Biosystems Co., Ltd., USA).

### Statistical analysis

Quanto software was used to estimate the adequate sample size for our study. The relative risk was set to 1.30 and the statistical power was more than 80%. Demographic and clinical data between sepsis cases and controls were compared by chi-square test (χ^2^ test) and by Student's t-test. The differences in genotype and allele frequencies of *TLR4* and *CD14* were compared among the groups using the χ2 test and when appropriate, Fisher’s exact test (two-sided analysis) was used as indicated. Odds ratios (OR) and 95% confidence intervals (CIs) were calculated to assess the relative risk conferred by a particular allele and genotype. Hardy-Weinberg equilibrium analysis was tested by comparing the detected genotype distribution with the theoretical distribution estimated on the basis of the allele frequencies in the control group (Table 3). The linkage disequilibrium (LD) among SNPs of the *TLR4* gene or the *CD14* gene was examined by pair-wise comparisons of D’ using Haploview, version 4.1. The haplotypes and their frequencies were estimated based on a Bayesian algorithm using the Phase program [29]. No correction for multiple testing was performed during analysis of the data. All reported *p* values were two-tailed and *p* <0.05 was considered to be significant. The SPSS statistical software package version 13.0 was used for statistical analysis.

**Table 3:** **Hardy-Weinberg equilibrium analysis**

**Polymorphism**	**Actual distribution**			**Theoretical distribution**			***χ2***	***P***
	**Genotypes**			**Genotypes**				
TLR4								
	CC	CT	TT	CC	CT	TT		
rs10759932(T/C)	7	62	130	7.26	61.49	130.26	0.014	0.993
	AA	GA	GG	AA	GA	GG		
rs12377632(C/T)	19	94	86	21.89	88.22	88.89	0.854	0.653
	CC	CT	TT	CC	CT	TT		
rs1927907(C/T)	136	47	5	135.32	48.36	4.32	0.148	0.928
	AA	GA	GG	AA	GA	GG		
rs11536879(A/G)	145	48	3	145.72	46.56	3.72	0.187	0.911
	CC	GC	GG	CC	GC	GG		
rs11536889(G/C)	15	75	109	13.85	77.30	107.85	0.176	0.916
	CC	CG	GG	CC	CG	GG		
rs7873784(G/C)	2	39	157	2.33	38.33	157.33	0.060	0.970
CD14								
	AA	GA	GG	AA	GA	GG		
rs2569190(A/G)	65	87	45	59.76	97.48	39.76	2.279	0.320
	AA	CA	CC	AA	CA	CC		
rs2563298(C/A)	4	53	141	4.70	51.60	141.70	0.145	0.930

## Results

One hundred fifty-two patients with sepsis and one hundred ninety-nine control subjects were genotyped. The genotypic and allelic frequencies for SNPs are shown in Table 4. The frequency of all genotypes studied did not reveal any difference using the Hardy-Weinberg equilibrium analysis in control groups (*p* > 0.05).

**Table 4:** **The genotype and allele frequencies of patients with sepsis and controls**

**Polymorphism**	**Genotypes**			***χ2***	***P***	**Alleles**		***OR(95% CI)***	***P***
	**11**	**12**	**22**			**1**	**2**		
TLR4									
rs10759932(T/C)	TT	TC	CC			T	C		
Controls	130	62	7	0.987	0.611	322	76	0.874(0.592-1.290)	0.499
Cases	106	40	6			252	52		
rs12377632(C/T)	CC	CT	TT			C	T		
Controls	86	94	19	0.433	0.805	266	132	0.916(0.665-1.261)	0.591
Cases	71	67	14			209	95		
rs1927907(C/T)	CC	CT	TT			C	T		
Controls	136	47	5	0.499	0.802	319	57	1.075(0.710-1.630)	0.732
Cases	109	37	6			255	49		
rs11536879(A/G)	AA	AG	GG			A	G		
Controls	145	48	3	0.756	0.72	338	54	1.031(0.670-1.588)	0.889
Cases	110	41	1			261	43		
rs11536889(G/C)	GG	GC	CC			G	C		
Controls	109	75	15	6.918	0.031	293	105	1.430(1.032-1.981)	0.031
Cases	62	77	13			201	103		
rs7873784(G/C)	GG	GC	CC			G	C		
Controls	157	39	2	0.616	0.752	353	43	1.173(0.737-1.866)	0.501
Cases	116	34	2			266	38		
CD14									
rs2569190(A/G)	AA	AG	GG			A	G		
Controls	65	87	45	3.973	0.137	217	177	1.322(0.975-1.793)	0.073
Cases	58	72	22			188	116		
rs2563298(C/A)	CC	CA	AA			C	A		
Controls	141	53	4	12.41	0.001	335	61	2.454(1.458-4.130)	0.001
Cases	132	19	1			283	21		

No correction for multiple testing was performed during analysis of the data.

As shown in Table 4, a statistical significance was observed between rs11536889 in *TLR4* gene and rs2563298 in *CD14* gene in patients with sepsis and controls. The genotype frequency of rs11536889 indicated a trend of higher frequency of GC + CC in the sepsis patients (*p = 0.009*<*0.05*), [*OR* and *95% CI: 1.758*(*1.147-2.695*)]. The C allele was significantly associated with susceptibility to sepsis (*p = 0.031*<*0.05*) [*OR* and *95% CI: 1.430* (*1.032-1.981*)]. Compared to healthy controls, the rs2563298 was significantly associated with a risk of sepsis, and the frequencies of the CC, CA and AA genotypes of rs2563298 were 86.8%, 12.5% and 0.7% in cases of sepsis and were 71.2%, 26.8% and 2.0% in control subjects, respectively. The C allele was associated with a significantly increased risk of sepsis as compared with the A allele (*p =* 0.001<*0.05*) [*OR* and *95% CI: 2.454* (*1.458-4.130*)].

**Figure 1:**
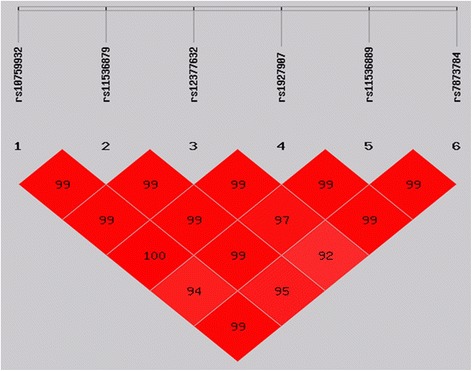
**Linkage disequilibrium plot of six SNPs of the** ***TLR4*** **gene in patients with sepsis.** D’ corresponding to each SNP pair is expressed as a percentage and shown within the respective squares. Higher D’ is indicated by a brighter red colour.

Figure 1 shows the pair-wise LD of the SNPs of *TLR4* and *CD14* genes in patients with sepsis. The haplotype frequency of the eight SNPs of *TLR4* gene and two SNPs of *CD14* gene were estimated. For the *TLR4* gene, there were eleven SNP haplotypes detected, and only four had frequencies greater than 5% (Table 5). As shown in Table 5, the results indicate that one haplotype (TACCCG) contributed to a significant difference (*p = 0.006*<*0.05*) [*OR* and *95% CI: 1.590* (*1.143-2.211*)], which therefore shows that this haplotype serves as an increase risk factor for sepsis. The other haplotypes were not associated with susceptibility to sepsis. As for the *CD14* gene, there were four SNP haplotypes detected (Table 6). Compared to controls, the haplotype AG was significantly different (*p = 0.001*<*0.05*) [*OR* and *95% CI: 0.410* (*0.244-0.690*)], and this seems to provide a protective role in sepsis.

**Table 5:** **Haplotype distribution of TLR4 in patients with sepsis and controls**

**Haplotypes**	**Cases**	**Controls**	***χ2***	***P***	**OR (95%)**
CATTGG	49	55	0.722	0.395	1.198(0.789-1.820)
TACCCG	103	97	7.651	0.006	1.590(1.143-2.211)
TACCGG	104	152	1.179	0.278	0.842(0.616-1.149)
TGTCGC	36	38	0.962	0.327	1.273(0.785-2.062)
TGTCGG	7	13	0.578	0.447	0.698(0.275-1.771)
CATCCG	0	1	-	-	-
CATCGG	3	10	-	-	-
CATTCG	0	0	-	-	-
TACCGC	2	1	-	-	-
TATCGG	0	1	-	-	-
TGTCCC	0	0	-	-	-

From left to right: rs10759932 T/C; rs11536879 A/G; rs12377632 C/T; rs1927907 C/T; rs11536889 G/C and rs7873784 G/C.

**Table 6:** **Haplotype distribution of CD14 in patients with sepsis and controls**

Haplotypes	Cases	Controls	*χ2*	*P*	*OR(95%)*
AG	21	61	11.841	0.001	0.410(0.244-0.690)
CA	188	217	3.783	0.052	1.252(0.997-1.832)
CG	95	116	0.363	0.547	1.105(0.798-1.529)
AA	-	-	-	-	-

From left to right: rs2569190 A/G and rs2563298 C/A.

## Discussion

In this study, we investigated the association of *TLR4* gene and *CD14* gene SNPs (*TLR4*: rs10759932, rs11536889, rs7873784, rs12377632, rs1927907, rs1153879 and *CD14*: rs2563298, rs2569190) with susceptibility to sepsis. Our results demonstrate that statistically significant associations with risk of sepsis were observed for the candidate rs11536889 SNP of *TLR4* gene, rs2563298 SNP of *CD14* gene and two haplotypes.

TLR4 is a type I transmembrane protein which plays a key role in host defense mechanisms by activating both innate and adaptive immunity against pathogenic microbial infections such as Escherichia coli, Klebsiella pneumoniae, Staphylococcus aureus, Acinetobacter baumannii and Pseudomonas aeruginosa [30,31]. It plays an important role in some immune and inflammatory diseases. Several studies have reported a functional significance of *TLR4* gene polymorphisms. Previous studies had reported that two SNPs of the *TLR4* gene, which are located in promoter region, could lead to hyporesponsiveness to lipopolysaccharide (LPS) [32,33]. The possible involvement of these SNPs in the development of sepsis has been widely reported [17,24,25,34]. However, the distribution of these SNPs is rare in the Chinese [35,36] as well as in the general Asian population. Our results show that frequency of the GC + CC genotype rs11536889, which is located in the 3’-untranslated region, is higher in the sepsis group when compared to the controls. With the present study, there is no evidence that rs10759932, rs7873784, rs12377632, rs1927907 and rs1153879 are associated with susceptibility to sepsis. By haplotype analysis, it was found that the TACCCG haplotype was associated with a significantly increased risk of sepsis when compared with the control group.

Fukusaki *et al.* [37] suggested that CC genotype rs11536889 was associated with moderate and severe periodontitis in the Japanese population. Hishida *et al.* [38,39] have shown that GC + CC genotype rs11536889 may be associated with severe gastric atrophy related to *Helicobacter pylori* infection. Zhou *et al.* [40] examined whether polymorphisms of the *TLRs* genes were associated with hepatitis B virus recurrence after liver transplantation. They showed a significant association of one SNP, namely rs11536889, with hepatitis B virus recurrence after liver transplantation. Hepatitis B virus recurrence after liver transplantation was higher than in the patients with the CC genotype when compared to other genotypes. Miedema *et al.* [41] found that rs11536889 polymorphism may be associated with an increased risk of developing chemotherapy-induced neutropenia. Sato *et al*. [21] investigated whether polymorphisms of rs11536889 were associated with expression or function of *TLR4*. They found that *TLR4* mRNA expression in PBMCs among GG, GC and CC genotypes did not significantly respond to LPS stimulation. However, the TLR4 protein expressed at the cell surface membrane was different. Therefore, it is possible that the G allele of rs11536889 may inhibit translation rather than gene transcription in order to regulate the expression of the *TLR4* gene. Meanwhile, this study shows that certain genotypes may affect the release of inflammatory cytokines. As shown by Mansur *et al*., polymorphism of rs11536889 can affect the clinical outcome of patients with sepsis. In addition, the G allele of rs11536889 may increase the incidence of gram-negative infections [42].

*CD14* is also an important recognition receptor which plays a key role in the immune and inflammatory responses. Previous studies have shown that polymorphisms of *CD14* gene are involved with some inflammatory diseases, such as asthma [43], inflammatory bowel disease [44,45], ulcerative colitis [44] and Crohn's disease [46]. Currently, most studies focused on the SNPs which were located within *CD14* gene promoter region. Results from previous studies have shown that these SNPs were associated with the prognosis of critically ill patients [47,48]. Studies have shown that genetic polymorphisms of these SNPs can affect *CD14* gene expression as well as the level of soluble CD14 in serum [22,23], and that this might be involved with the occurrence of sepsis and the subsequent prognosis of the disease [26,28]. However, these studies are not consistent. One meta-analysis evaluated the associated between *CD14* promoter -159C/T polymorphism and the risk of sepsis. The results demonstrated that this SNP is unlikely to be a risk factor for susceptibility to sepsis. Only a weak correlation was found in the Asian population [27]. This study also illustrates the importance of genetic background and environmental factors in establishing whether SNPs may be risk factors for susceptibility to sepsis.

Our results suggest that there is no evidence that rs2569190 is associated with susceptibility to sepsis. Compared to the control group, CC genotypes of rs2563298 in the sepsis group were significantly higher and the frequency of C allele in the sepsis group was also significantly higher. By haplotype analysis, it was found that the AG haplotype may be a protective factor to sepsis. Rs2563298 is located in the 3’-untranslated region of the *CD14* gene. This study is the first to assess the potential implications of *CD14* gene rs2563298 on susceptibility to sepsis. Previous studies suggested that SNPs in the 3’-untranslated region may have an important role in mRNA translation. Liu *et al.* [49] suggested that *CD14* genetic polymorphisms may affect the length of *CD14* transcripts or the efficiency protein translation, which could influence the function of *CD14* and lead to a dysregulation of the immune response.

SNPs in the 3’-untranslated region probably have no direct effect on the primary structure (ie. amino acid sequence) of the protein. But the SNPs may change the function of 3’-end of mRNA or affect the mRNA stability in order to regulate target gene expression which may, in turn, affect the process of transmembrane conductance signals.

The observed association of higher susceptibility to sepsis among TLR4 rs11536889 and *CD14* rs2563298 genotypes may help intensive care specialists to identify patients at risk of developing sepsis.

## Conclusions

In summary, this study indicates that SNPs (*TLR4*: rs 11536889 and *CD14*: rs2563298) and two haplotypes are associated with susceptibility to sepsis in a Chinese population.

## Abbreviations

*CD14*: Cluster of differentiation 14
PCR: Polymerase chain reaction
SNPs: Single nucleotide polymorphisms
*TLR4*: Toll-like receptor 4
